# Solvent Evaporation-Controlled Stereocomplexation in PLLA/PDLA Films for Sustainable Packaging

**DOI:** 10.3390/polym18111285

**Published:** 2026-05-24

**Authors:** Yottha Srithep, Tamilselvan Mohan, Arissara Phosanam, Rupert Kargl, Karin Stana Kleinschek

**Affiliations:** 1 Manufacturing and Materials Research Unit, Department of Manufacturing Engineering, Faculty of Engineering, Mahasarakham University, Mahasarakham 44150, Thailand; 2 Institute of Chemistry and Technology of Biobased System (IBioSys), Graz University of Technology, Stremayrgasse 9, 8010 Graz, Austria; rupert.kargl@tugraz.at (R.K.); karin.stanakleinschek@tugraz.at (K.S.K.); 3 Department of Food Technology and Nutrition, Faculty of Natural Resources and Agro-Industry, Kasetsart University, Sakon Nakhon 47000, Thailand; arissara.p@ku.th

**Keywords:** stereocomplex polylactide, PLLA/PDLA blends, solvent evaporation, crystallization behavior, barrier properties, microbial resistance, biodegradable polymers

## Abstract

The formation of stereocomplex (SC) crystallites in poly(L-lactide) (PLLA)/poly(D-lactide) (PDLA) blends has attracted significant attention due to its potential to enhance the performance of biodegradable polymer films. In this study, the effect of solvent evaporation kinetics on the crystallization behavior, microstructure, and functional properties of PLLA/PDLA blend films was systematically investigated. Films with various blend ratios were prepared under open-lid (fast evaporation) and closed-lid (slow evaporation) conditions. Differential scanning calorimetry (DSC), wide-angle X-ray diffraction (WAXD), and small-angle X-ray scattering (SAXS) analyses revealed that slow solvent evaporation significantly promotes stereocomplex formation, particularly at the equimolar (50:50) composition, resulting in a higher degree of crystallinity and a more compact structure compared to fast evaporation conditions. These structural changes were directly correlated with improved functional properties. The optimized PLLA/PDLA (50:50) films exhibited a substantial reduction in water vapor permeability from 22.7 to 3.11 g·mm/m^2^·day·kPa (~86% decrease) and a marked decrease in microbial growth, as evidenced by reduced total plate count (TPC) values compared to neat polymers. The enhanced barrier performance and reduced microbial proliferation were attributed to the reduced free volume and increased tortuosity associated with densely packed stereocomplex crystallites, as supported by DSC and WAXD results. These findings demonstrate the importance of solvent evaporation kinetics in tailoring structure–property relationships to control stereocomplex formation and multiscale structural organization, providing a practical strategy for biodegradable packaging films.

## 1. Introduction

Poly(lactic acid) (PLA) has emerged as one of the most promising biodegradable polymers for sustainable packaging applications due to its renewable origin, biocompatibility, and processability [1,2,3]. However, its relatively low thermal resistance, brittleness, and moderate barrier performance toward gases and moisture have limited its broader industrial utilization, particularly in food packaging [4].

One of the most effective strategies to enhance the performance of PLA is the formation of stereocomplex (SC) crystallites through the interaction between poly(L-lactide) (PLLA) and poly(D-lactide) (PDLA) [5,6]. Compared to conventional homocrystals (HCs), SC crystallites exhibit a higher melting temperature, improved thermal stability, and enhanced mechanical properties due to the strong intermolecular interactions between enantiomeric chains [7]. As a result, SC-PLA materials have attracted considerable attention for advanced packaging and biomedical applications [8].

Previous studies have extensively investigated stereocomplex formation in PLA systems, primarily focusing on parameters such as blend composition, molecular weight, and processing techniques, including melt blending and thermal annealing [9,10]. These approaches have successfully enhanced SC crystallization; however, they predominantly rely on thermal control and equilibrium crystallization conditions. In contrast, the influence of solvent evaporation kinetics during solution casting—one of the most widely used techniques for thin-film fabrication—remains insufficiently explored [11]. Unlike melt processing, solvent evaporation introduces non-equilibrium conditions, where chain mobility, diffusion, and intermolecular interactions are dynamically governed by solvent removal rate. As a result, evaporation kinetics can significantly influence the competition between stereocomplex (SC) and homocrystal (HC) formation, yet this effect has not been systematically quantified or clearly linked to functional properties. This gap critically limits the ability to rationally design PLA films with optimized barrier and microbial resistance. While previous studies have reported improved crystallinity in SC-PLA systems, the direct correlation between processing-induced crystallization pathways and functional properties such as permeability and antimicrobial behavior remains insufficiently understood. To the best of our knowledge, a systematic investigation linking solvent evaporation kinetics to both stereocomplex crystallization and functional performance in PLA films has not been established or quantitatively demonstrated [12].

In solution-cast systems, solvent removal governs chain mobility, diffusion, and intermolecular interactions, which are critical factors in determining crystallization pathways [13]. Rapid solvent evaporation may restrict chain rearrangement and induce phase separation, whereas slow evaporation could allow sufficient time for ordered SC formation [12]. Furthermore, in non-equimolar PLLA/PDLA blends, excess polymer chains may not fully participate in stereocomplexation, potentially leading to unique structural states such as kinetically trapped amorphous regions [14].

Although PLLA/PDLA stereocomplex systems have been extensively studied, previous studies mainly focused on composition effects and thermal processing conditions. Limited attention has been paid to how solvent evaporation kinetics govern hierarchical structural evolution and its correlation with multifunctional properties in solution-cast PLLA/PDLA films. In particular, the relationships among evaporation-controlled crystallization behavior, nanoscale structural organization, barrier performance, and microbial proliferation remain insufficiently understood [13,15].

## 2. Materials and Methods

### 2.1. Materials

Poly(L-lactide) (PLLA, grade L175) and poly(D-lactide) (PDLA, grade D120) were both kindly supplied by Total Corbion PLA (Thailand). The selected PLLA and PDLA grades possessed relatively comparable molecular weights and narrow polydispersity indices (PDIs), which are favorable for stereocomplex formation and reproducible crystallization behavior. Chloroform (CHCl_3_, analytical grade, used as received (≥99.8% purity)) was purchased from Fisher Scientific, Waltham, MA, USA, and used without further purification.

The number-average molecular weight (Mn), weight-average molecular weight (Mw), and polydispersity index (PDI) of PLLA and PDLA were determined using gel permeation chromatography (GPC) with chloroform as the eluent at 35 °C and polystyrene standards for calibration. The results were as follows:

– PLLA (L175): Mn = 114,000 g/mol, Mw = 210,000 g/mol, PDI = 1.84;
– PDLA (D120): Mn = 90,000 g/mol, Mw = 152,000 g/mol, PDI = 1.68.

### 2.2. Sample Preparation

PLLA and PDLA were dissolved in chloroform at a total polymer concentration of 10 wt%, and the mixture was stirred for 2 h at room temperature to ensure homogeneity. The blended solutions were prepared at five different PLLA:PDLA weight ratios: 100:0 (PLLA), 70:30, 50:50, 30:70, and 0:100 (PDLA). Each solution was poured into a clean Petri dish and allowed to dry slowly in a closed-lid condition at room temperature (~25 °C) for approximately 3 days to allow for controlled solvent evaporation and crystallization. All film preparations and characterizations were independently repeated under identical experimental conditions to ensure reproducibility.

Unless otherwise noted, all films used in the subsequent characterizations (optical, mechanical, AFM, WVP, etc.) were prepared under these conditions. For comparative purposes, all PLLA/PDLA compositions were prepared under both open-lid and closed-lid conditions for comparative evaluation of solvent evaporation effects, as discussed in Section 3.1.

### 2.3. Material Characterization

#### 2.3.1. Tensile Testing

Tensile properties of the PLLA/PDLA films were evaluated using a universal testing machine (Shimadzu AGS-X 5, Shimadzu Corporation, Kyoto, Japan). Dog-bone-shaped specimens were cut from solvent-cast films using a DIN 53504 S3A die, with a gauge length of 20 mm and specimen thickness of approximately 0.4–0.5 mm. The tests were performed at room temperature using a constant crosshead speed of 5 mm/min. At least five specimens were tested for each composition to ensure statistical reliability. Tensile strength, elongation at break, and Young’s modulus were calculated from the resulting stress–strain curves.

#### 2.3.2. Polarized Optical Microscopy (POM)

The crystalline morphology of PLLA, PDLA, and PLLA/PDLA blend films was observed using polarized optical microscopy (POM) under crossed polarizers. Small amounts of samples were placed on glass slides, heated until complete melting, and subsequently cooled slowly to room temperature to allow for crystal formation. Representative images were recorded to evaluate the development of homocrystalline and stereocomplex crystalline structures.

#### 2.3.3. Fourier Transform Infrared Spectroscopy (FTIR)

FTIR spectra of the films were recorded using a Fourier transform infrared spectrometer in the wavelength range of 4000–600 cm^−1^ at room temperature. The measurements were performed in ATR mode, and the film samples were directly placed on the ATR crystal without KBr pellet preparation or additional sample treatment.

#### 2.3.4. Atomic Force Microscopy (AFM)

Surface morphology of the PLLA/PDLA blend films was analyzed using an Atomic Force Microscope (AFM; Tosca 400, Anton Paar GmbH, Graz, Austria) operated in tapping mode. All measurements were conducted under ambient conditions. Samples were mounted on the sample stage without additional coating. A scan area of 5 × 5 μm^2^ was typically used. The resulting topography images were used to evaluate surface roughness and phase separation characteristics. At least three independently measured regions were analyzed for each sample to ensure reproducibility and statistical reliability.

#### 2.3.5. Simultaneous SAXS/WAXS Analysis

The crystalline structure and nanostructural organization of the films were analyzed using a SAXSpoint 2.0 system (Anton Paar GmbH, Graz, Austria). The measurements were conducted in transmission mode at room temperature.

The system utilizes a microfocus Cu Kα X-ray source (*λ* = 0.15418 nm) operated at 50 kV and 1 mA. Small-angle X-ray scattering (SAXS) and wide-angle X-ray scattering (WAXS) data were simultaneously collected from each sample in a single run. Scattering profiles were recorded as intensity versus the scattering vector *q*, calculated by the equation:

(1) $$q=\frac{4\pi }{\lambda }sin(\theta )$$

where *λ*\lambda is the X-ray wavelength and *θ*\theta is the scattering angle. The *q*-range for SAXS was 0.05–1.0 nm^−1^, corresponding to structural features in the 6–125 nm range. WAXS data covered the equivalent 2*θ* range of approximately 5° to 30°, sufficient for identifying characteristic peaks of PLA homocrystals and stereocomplex crystallites [16].

All data were processed using instrument-specific software provided by Anton Paar. The d-spacings were calculated using d = 2*π*/*q*, and phase identification was based on previously reported crystallographic assignments for PLA systems.

#### 2.3.6. Differential Scanning Calorimetry (DSC)

Thermal properties of PLLA, PDLA, and PLLA/PDLA blend films were characterized using differential scanning calorimetry (DSC 4000, PerkinElmer, Waltham, MA, USA). Samples with a mass of approximately 4–5 mg were sealed in aluminum pans and heated from 0 to 250 °C at a heating rate of 10 °C/min under a nitrogen atmosphere.

The homocrystal melting temperature (T_m,HC_), and stereocomplex melting temperature (T_m,SC_), along with their corresponding melting enthalpies (Δ*H_m,HC_* and Δ*H_m,SC_*), were determined from the heating scan. The degree of crystallinity for homocrystals (*X_c,HC_*) and stereocomplex crystals (*X_c,SC_*) was calculated using the following equations:

(2) $$X_{c,HC}(\%)=\frac{\Delta H_{m,HC}}{\Delta H_{m,HC}^{0}}\times 100$$

(3) $$X_{c,SC}(\%)=\frac{\Delta H_{m,SC}}{\Delta H_{m,SC}^{0}}\times 100$$

where $\Delta H_{m,HC}$ and $\Delta H_{m,SC}$ are the measured melting enthalpies of homocrystals and stereocomplex crystals, respectively. $\Delta H_{m,HC}^{0}$ (93 J/g) and $\Delta H_{m,SC}^{0}$ (142 J/g) represent the melting enthalpies of 100% crystalline PLA homocrystals and stereocomplex crystals, respectively [17,18]. The total crystallinity ($X_{c,total}$) was determined as:

(4) $$X_{c,total}(\%)=X_{c,HC}+X_{c,SC}$$

All DSC measurements were independently repeated at least three times to confirm reproducibility, and representative thermograms are presented.

#### 2.3.7. UV–Vis Spectroscopy

UV–Vis transmittance spectra of the films were measured using a UV–Visible spectrophotometer (Lambda 12, PerkinElmer, Waltham, MA, USA) in the wavelength range of 200–800 nm at room temperature. The films were directly placed in the sample holder without further treatment.

#### 2.3.8. Water Vapor Permeability (WVP) Measurement

The water vapor permeability (WVP) of the films was evaluated using a gravimetric method adapted from ASTM E96/E96M-16 (Desiccant Method; ASTM International, West Conshohocken, PA, USA, 2016), with minor modifications. Circular film specimens were sealed over the openings of pre-weighed glass vials (yogurt cups) containing anhydrous calcium chloride (CaCl_2_, RH ≈ 0%) using hot-melt adhesive and parafilm, followed by an external PLA film layer to ensure a vapor-tight seal [19].

The sealed vials were placed in a desiccator containing a saturated sodium chloride (NaCl) solution, which maintained a constant relative humidity of approximately 75% at room temperature (≈25 °C). The vials were left undisturbed, and the weight gain was recorded daily over a 7-day period to monitor water vapor transmission.

WVP was calculated using the following equation:

(5) $$WVP=\frac{WVTR\times L}{\Delta P}$$

where WVTR is the slope of the weight gain vs. time curve per unit area (g/m^2^·s), L is the film thickness (m), and Δ*P* is the partial pressure difference of water vapor across the film (Pa). A Δ*P* value of 2.375 kPa was used, corresponding to the vapor pressure difference between 75% RH (NaCl) and 0% RH (CaCl_2_) at 25 °C. All measurements were performed in triplicate, and the results are reported as the mean ± standard deviation.

#### 2.3.9. Degradation Experiment

The degradation behavior of PLLA/PDLA blend films was evaluated under both hydrolytic (alkaline) and enzymatic conditions. For alkaline degradation, specimens were immersed in 20 mL of aqueous NaOH solution at concentrations 1.0 M (3 h at 25 °C). After immersion, the samples were thoroughly rinsed with deionized water for 30 min to remove residual alkali and then dried to a constant weight under vacuum before further analysis [20].

The weight loss of each sample was determined gravimetrically according to:

(6) $$Weight\;loss(\%)=\frac{Wo-Wt}{Wo}\times 100$$

where Wo and Wt represent the initial and final dry weights of the samples, respectively.

#### 2.3.10. Total Plate Count (TPC) Analysis

For antimicrobial evaluation, film samples were incubated with microbial cultures under controlled conditions, and total plate count (TPC) analysis was performed after incubation at 37 °C for 24 h using the spread plate method.

*Sample preparation*

Fresh minced pork was packed in glass jars sealed with different PLA-based films (PLLA, PDLA, 50:50 blend, and 70:30 blend) and stored at 4 °C. Two sets of samples were used: (i) fresh pork at day 0 and (ii) pork after 6 days of storage. At each time point, 10 g of pork was aseptically collected from the jars, transferred into a sterile stomacher bag, and homogenized with 90 mL of sterile 0.85% NaCl solution to obtain a 10^−1^ dilution. Serial dilutions were then prepared up to 10^−6^ [21].

*Preparation of LB agar medium*

The LB agar medium was prepared using a standard formulation. For 1 L of medium, 5 g of yeast extract, 5 g of sodium chloride (NaCl), 10 g of peptone (soy-based), and 17 g of agar-agar were dissolved in distilled water. The pH of the solution was adjusted to approximately 7.0 using sodium hydroxide (NaOH). The prepared medium was sterilized by autoclaving at 121 °C for 15 min. After sterilization, the medium was allowed to cool to approximately 50–55 °C and then aseptically poured into sterile Petri dishes under a laminar flow hood. The agar plates were left to solidify at room temperature and subsequently stored at 4 °C until use.

*Colony counting*

From each dilution, 0.1 mL was spread-plated onto LB agar plates in triplicate. The plates were incubated at 37 °C for 24 h. Colonies in the range of 30–300 were counted and expressed as colony-forming units per gram of pork (CFU/g).

### 2.4. Statistical Analysis

All experiments were conducted independently at least three times unless otherwise specified. The reported values are presented as mean ± standard deviation. Statistical comparisons were performed using one-way analysis of variance (ANOVA) in OriginPro 2024 software (OriginLab Corporation, Northampton, MA, USA) and differences were considered statistically significant at *p* < 0.05. All instruments were calibrated and operated according to the manufacturers’ recommended procedures prior to measurements.

## 3. Results and Discussion

### 3.1. Effect of Solvent Evaporation Conditions on Film Morphology

**Figure 1:**
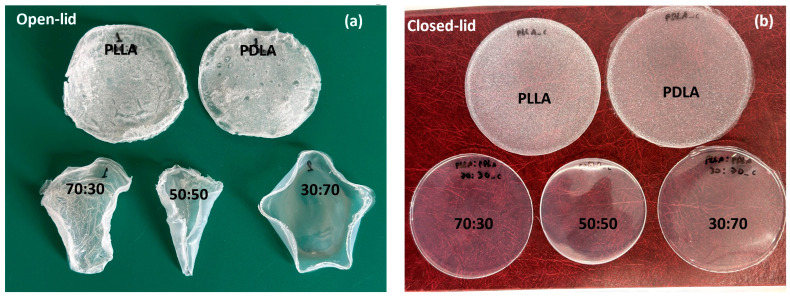
Visual comparison of solvent-cast PLLA/PDLA films prepared under (**a**) open-lid and (**b**) closed-lid solvent evaporation conditions at different blend ratios, demonstrating the influence of evaporation kinetics on film morphology and optical clarity.

The morphology and visual appearance of PLLA, PDLA, and PLLA/PDLA blend films were strongly influenced by solvent evaporation conditions, as illustrated in Figure 1. Distinct differences were observed between films prepared under open-lid (fast evaporation) and closed-lid (slow evaporation) conditions, highlighting the critical role of solvent removal kinetics in governing structural development. The average film diameter and thickness for each composition are summarized in Table 1.

All films were cast using the same volume of solution under identical closed-lid conditions. The reduction in film diameter for the 50:50 PLLA/PDLA blend is associated with the optimized formation of stereocomplex (SC) crystallites at the equimolar ratio. The strong intermolecular interactions between enantiomeric chains facilitate a more compact and densely packed structure, resulting in reduced free volume and enhanced shrinkage during solvent evaporation. During solvent evaporation, this compact structure undergoes greater shrinkage compared to homopolymer or non-equimolar blends, where chain pairing is incomplete. As a result, the 50:50 blend exhibits a smaller final diameter, reflecting its higher structural uniformity and packing efficiency.

Among the different compositions, the 50:50 PLLA/PDLA blend showed the highest optical clarity and most uniform morphology, indicating the most efficient stereocomplex formation at the equimolar ratio [22]. This observation is consistent with enhanced stereocomplex formation under closed-lid conditions. The improved homogeneity in this composition can be attributed to the balanced availability of PLLA and PDLA chains, which maximizes stereocomplexation and minimizes the presence of unpaired chains.

Under open-lid conditions, rapid solvent evaporation led to the formation of films with rough, irregular surfaces and noticeable opacity. The fast solvent removal restricted polymer chain mobility, hindering the orderly arrangement of PLLA and PDLA chains. As a result, the formation of well-defined stereocomplex (SC) crystallites was suppressed, and a higher degree of structural heterogeneity was observed. This is consistent with the limited time available for chain diffusion and intermolecular interactions, which are essential for stereocomplexation.

In contrast, films prepared under closed-lid conditions exhibited significantly improved transparency and smoother surface morphology. This morphological improvement is consistent with the reduced surface roughness observed by AFM analysis, where the RMS roughness (S_q_) decreased from 14.08 nm for neat PLLA and 15.72 nm for PDLA to 5.22 nm for the 50:50 PLLA/PDLA blend. The slower evaporation rate allowed sufficient time for chain rearrangement and diffusion, facilitating effective intermolecular interactions between PLLA and PDLA chains. This condition promoted the formation of more uniformly distributed SC crystallites, resulting in a denser and more homogeneous microstructure.

These results demonstrate that slower solvent evaporation promotes a denser and more homogeneous microstructure through enhanced stereocomplex formation. This behavior is quantitatively reflected by the reduced surface roughness, increased crystallinity, and improved barrier performance of the films [23].

**Figure 2:**
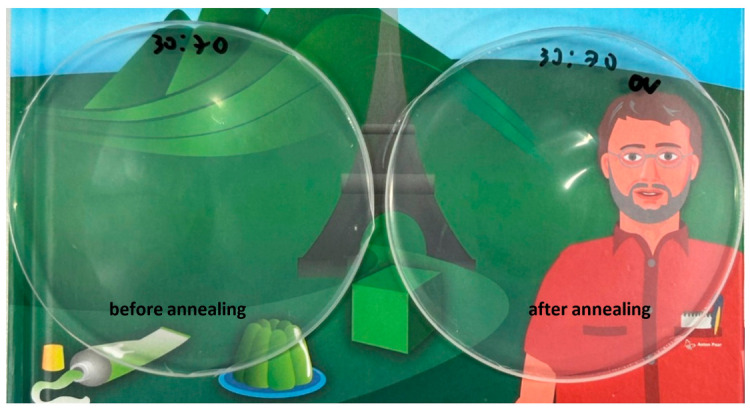
Optical appearance of PLLA/PDLA blend films (30:70) before (**left**) and after annealing at 100 °C for 30 min (**right**), showing no significant visual change in clarity or color.

To evaluate the thermal stability of the crystallized structures, selected PLLA/PDLA blend films were annealed at 100 °C for 30 min. No significant change in the optical appearance was observed after annealing, as shown in Figure 2. This suggests that the stereocomplex crystallites formed during solvent casting were thermally stable and retained their morphology under mild heat treatment. These morphological differences are expected to influence light scattering behavior, which directly affects the optical transparency of the films, as discussed in the following section.

### 3.2. UV–Vis Optical Properties

**Figure 3:**
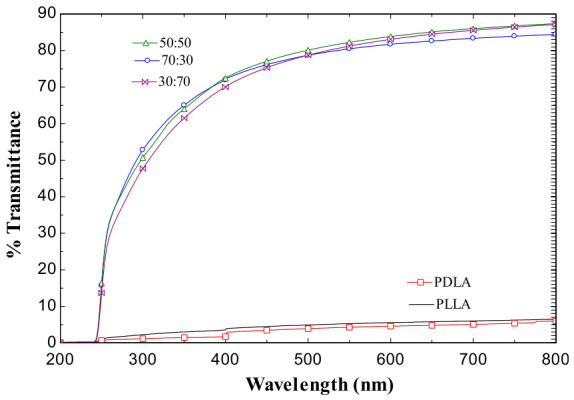
UV–Vis transmittance spectra of PLLA, PDLA, and PLLA/PDLA blend films with different weight ratios (70:30, 50:50, and 30:70). Measurements were independently repeated at least three times, and representative spectra are presented.

The UV–Vis transmittance spectra of PLLA, PDLA, and blends are presented in Figure 3. Both neat PLLA and PDLA films exhibited very low transmittance in the visible region, indicating poor optical clarity. In contrast, all PLLA/PDLA blend films showed significantly enhanced transparency, with transmittance values reaching approximately 80–88% at wavelengths above 500 nm [24].

Among the blends, the 50:50 composition exhibited the highest transmittance, followed by the 70:30 and 30:70 blends. The improved transparency of the blend films is attributed to the formation of stereocomplex (SC) crystallites, which promote more homogeneous molecular packing and reduce light scattering caused by large crystalline domains.

The superior transparency observed for the 50:50 blend suggests more efficient SC formation at near-equimolar composition, leading to a more uniform microstructure [22]. In contrast, the slightly lower transmittance of the 30:70 blend may be associated with excess PDLA chains, which can contribute to homocrystallization (HC) and phase heterogeneity, thereby increasing light scattering.

These results demonstrate that stereocomplex formation not only enhances thermal and mechanical properties but also significantly improves the optical transparency of PLA-based films, making them promising candidates for advanced packaging applications. To further understand the origin of the improved optical transparency, particularly in the equimolar blend, thermal analysis was performed to investigate the crystallization behavior and the formation of stereocomplex structures.

### 3.3. Thermal Behavior and Stereocomplex Formation (DSC)

**Figure 4:**
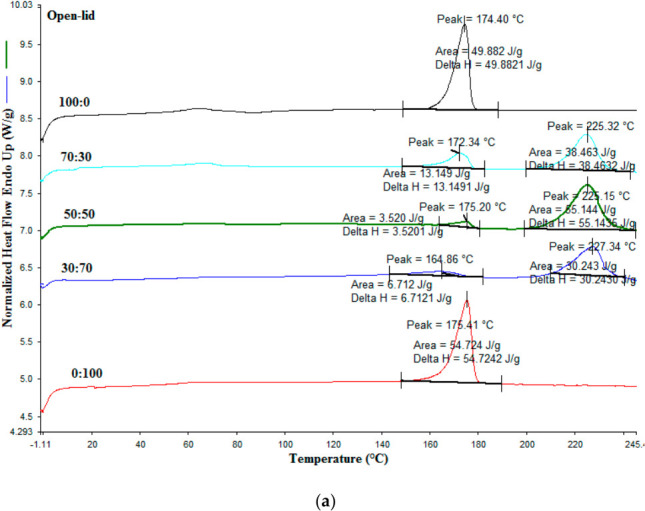

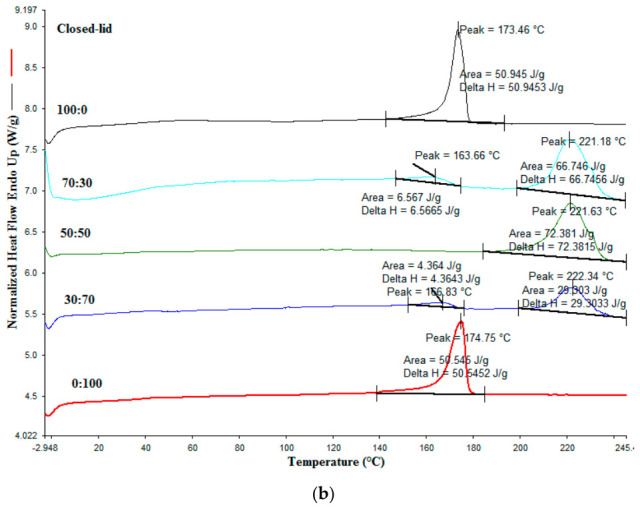
Representative DSC thermograms of PLLA, PDLA, and PLLA/PDLA blend films prepared under (**a**) open-lid (fast solvent evaporation) and (**b**) closed-lid (slow solvent evaporation) conditions.

The thermal behavior of PLLA, PDLA, and PLLA/PDLA blend films prepared under different solvent evaporation conditions was analyzed by DSC, as presented in Figure 4, with the corresponding thermal parameters summarized in Table 2. Clear differences were observed between films prepared under open-lid (fast evaporation) and closed-lid (slow evaporation) conditions, highlighting the critical role of solvent evaporation kinetics in governing crystallization behavior. No obvious glass transition (T_g_) or cold crystallization peaks were observed in the DSC thermograms, likely due to the high crystallinity and restricted chain mobility induced by stereocomplex formation.

Under open-lid conditions, all blend compositions exhibited multiple melting peaks in the temperature range of approximately 160–230 °C (Figure 4a). The lower-temperature peaks (~160–180 °C) are attributed to the melting of homocrystals (HC), while the higher-temperature peaks (~220–230 °C) correspond to stereocomplex (SC) crystals [25,26]. The coexistence of HC and SC phases indicates that rapid solvent evaporation restricts chain mobility and limits the extent of stereocomplexation, resulting in mixed crystalline structures. As shown in Table 2, the 50:50 blend exhibited the highest SC crystallinity (*X_c,SC_* = 38.80%), although a noticeable fraction of HC (*X_c,HC_* = 3.78%) was still present, suggesting incomplete stereocomplex formation under fast evaporation conditions.

In contrast, films prepared under closed-lid conditions showed significantly different thermal behavior (Figure 4b). The DSC thermograms were dominated by a single high-temperature melting peak (~221–222 °C), particularly for the 50:50 composition, indicating the preferential formation of stereocomplex crystals. The near disappearance of HC melting peaks confirms that slower solvent evaporation provides sufficient time for polymer chain diffusion and enantiomeric interactions, thereby promoting efficient stereocomplexation. This is further supported by the quantitative results in Table 2, where the SC crystallinity increased substantially under closed-lid conditions (e.g., *X_c,SC_* = 50.97% for the 50:50 blend), while HC crystallinity was largely suppressed.

For non-equimolar compositions (70:30 and 30:70), SC crystallization was still observed under both conditions; however, the extent of stereocomplex formation was lower compared to the equimolar blend. Under open-lid conditions, these compositions exhibited relatively higher HC content, indicating that excess PLLA or PDLA chains tend to crystallize into homocrystals due to insufficient pairing between enantiomeric chains. Under closed-lid conditions, although SC formation was enhanced (e.g., *X_c,SC_* = 47.00% for 70:30), residual HC contributions remained, reflecting the imbalance in chain stoichiometry.

The DSC results clearly indicate that solvent evaporation kinetics govern the competition between homocrystallization and stereocomplex crystallization. The enhanced SC formation under closed-lid conditions not only is reflected by the disappearance of HC melting peaks, but also directly correlates with the improved transparency, smoother surface morphology, and enhanced barrier properties observed in later sections. These findings suggest that controlled solvent evaporation promotes hierarchical structural organization that ultimately determines the functional performance of the films.

**Figure 5:**
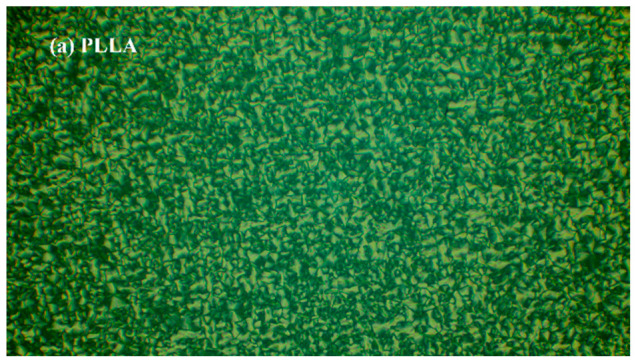

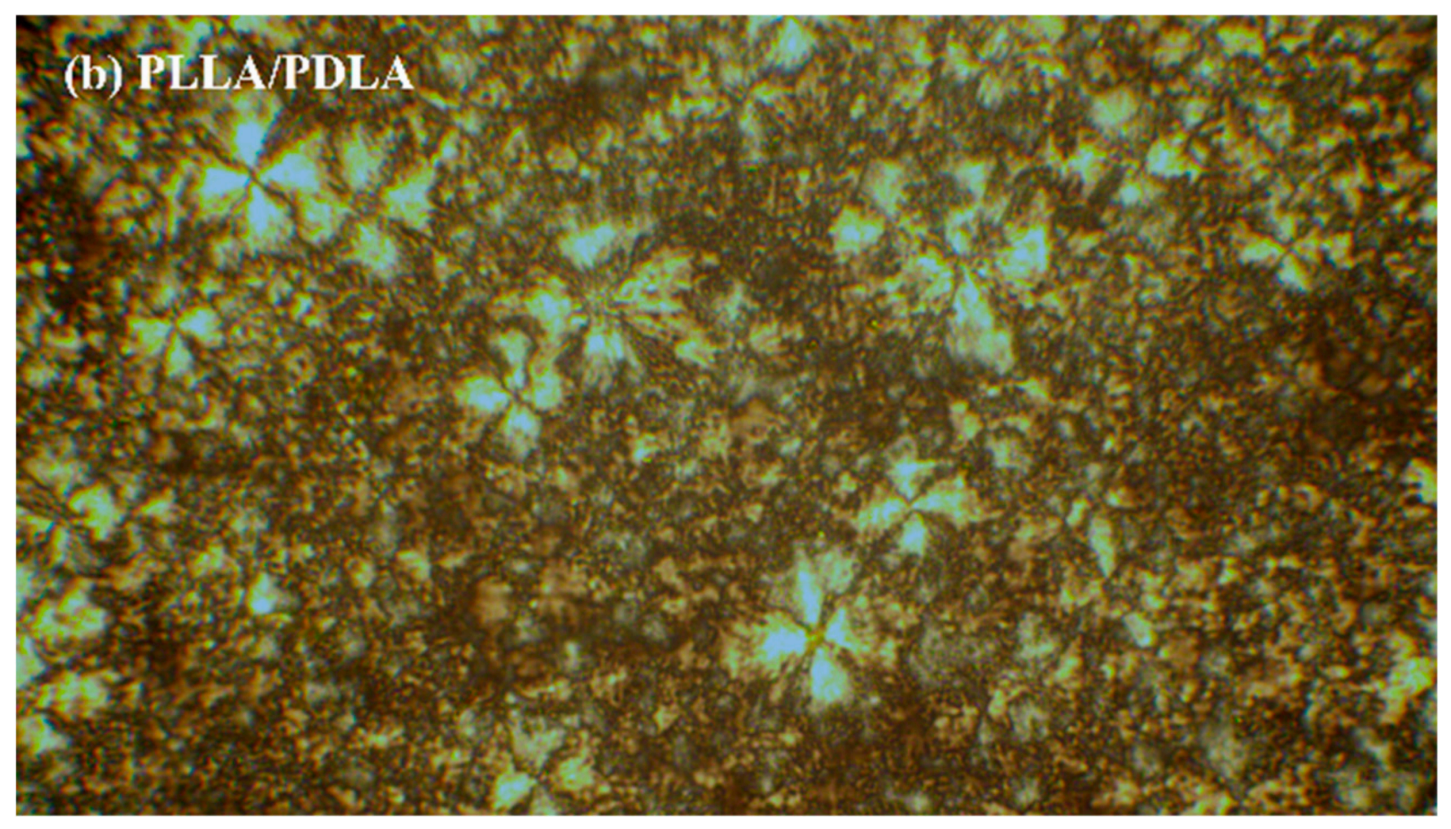
Polarized optical microscopy (POM) images of (**a**) PLLA and (**b**) PLLA/PDLA (50:50) blend films, highlighting the formation of stereocomplex crystallites in the blend compared to the homocrystalline structure of neat PLLA.

The crystalline morphology of the films was further examined using polarized optical microscopy (POM), as shown in Figure 5. The neat PLLA film exhibited a relatively uniform and fine texture, characteristic of homocrystalline structures. In contrast, the 50:50 PLLA/PDLA blend showed distinct bright crystalline domains, indicating the formation of stereocomplex (SC) crystallites. The presence of these well-defined structures suggests enhanced intermolecular interactions between PLLA and PDLA chains, leading to a more organized crystalline morphology [27]. These observations are consistent with the DSC results and further confirm the formation of SC structures in the equimolar blend, which contributes to the improved optical transparency and barrier properties of the films.

### 3.4. Surface Morphology Analysis by AFM

**Figure 6:**
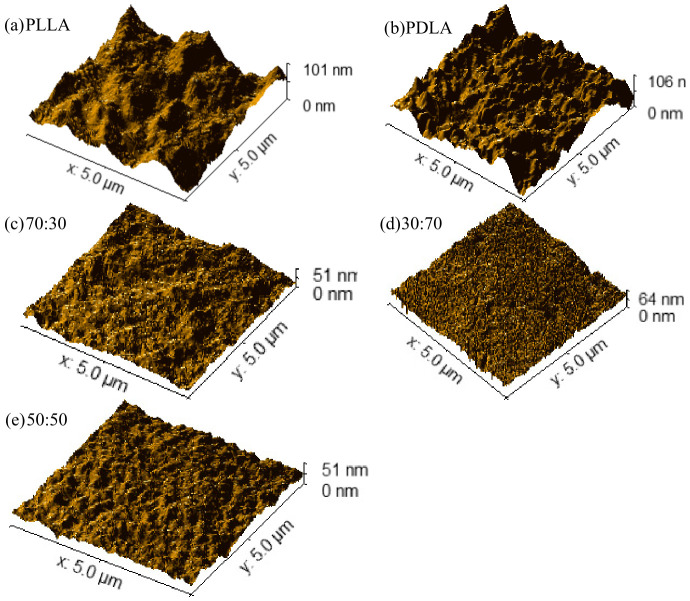
AFM 3D topographic images of PLLA, PDLA, and PLLA/PDLA blend films with different ratios (scan area: 5 µm × 5 µm), showing surface morphology variations influenced by blend composition.

The surface topography of PLLA/PDLA blend films was further investigated using atomic force microscopy (AFM), as presented in Figure 6. The 3D AFM images reveal the nanoscale surface morphology of neat PLLA, PDLA, and blends at various weight ratios (70:30, 50:50, and 30:70), with corresponding roughness parameters summarized in Table 3.

Neat PLLA and PDLA films exhibited relatively high surface roughness, with RMS roughness (Sq) values of 14.08 nm and 15.72 nm, respectively. These values reflect a more irregular surface, which is likely due to predominant homocrystalline (HC) structures and less compact molecular packing.

In contrast, the blend films, particularly the 50:50 PLLA:PDLA composition, showed significantly smoother surfaces, with an Sq value of only 5.22 nm. This dramatic reduction in surface roughness correlates with enhanced stereocomplex (SC) formation, which facilitates uniform molecular packing and suppresses the formation of coarse crystalline domains. Similarly, the 70:30 and 30:70 blends also showed reduced roughness compared to the neat components, although their values were slightly higher than that of the 50:50 blend.

These results suggest that the formation of stereocomplex crystallites leads to a more uniform and compact film structure, as reflected by the smoother surface morphology. The smoother surfaces are also consistent with the increased transparency observed under closed-lid casting conditions (Figure 1), supporting the hypothesis that SC crystallization contributes to improved film uniformity.

The reduced surface roughness observed under closed-lid conditions suggests enhanced structural uniformity associated with stereocomplex-rich domains. This compact morphology is expected to reduce diffusion pathways and contribute to improved barrier performance.

### 3.5. WAXD and SAXS Analysis

**Figure 7:**
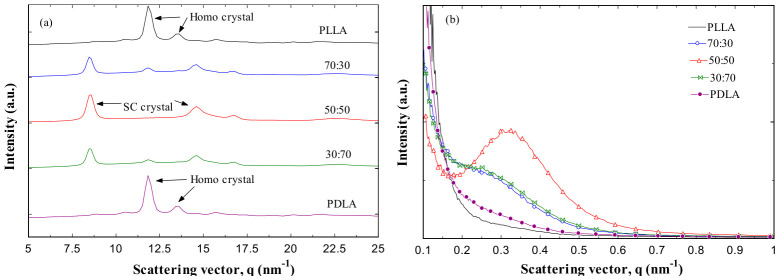
Representative WAXD and SAXS profiles, showing enhanced stereocomplex crystallization and nanoscale structural organization in the 50:50 blend. (**a**) WAXD patterns showing SC and HC crystal peaks in PLLA, PDLA, and blends. (**b**) SAXS profiles revealing nanoscale structural differences among compositions.

The crystalline structures of neat PLLA, PDLA, and blends were further analyzed by wide-angle X-ray diffraction (WAXD) and small-angle X-ray scattering (SAXS), as shown in Figure 7, with the corresponding diffraction parameters summarized in Table 4.

As presented in Figure 7a, neat PLLA and PDLA films exhibited characteristic diffraction peaks at 2*θ* ≈ 16.6° and 18.8°, corresponding to the (200)/(110) and (203) planes of the α-form homocrystalline (HC) phase. These values are consistent with the d-spacing values listed in Table 4 (1.80 and 1.51 nm), confirming the formation of conventional PLA homocrystals [28,29].

In contrast, the PLLA/PDLA blends, particularly the equimolar 50:50 composition, showed distinct diffraction peaks at 2*θ* ≈ 11.9° and 20.7°, corresponding to d-spacings of 2.03 and 1.00 nm, respectively (Table 4). These peaks are characteristic of the (110) and (300) planes of stereocomplex (SC) crystallites, indicating strong intermolecular interactions between PLLA and PDLA chains. The significantly higher intensity of these SC peaks for the 50:50 blend confirms the most efficient stereocomplexation at the equimolar ratio [14].

For non-equimolar blends (70:30 and 30:70), SC diffraction peaks were still observed in Figure 7a, but with reduced intensity compared to the 50:50 system. This suggests partial stereocomplex formation, where excess PLLA or PDLA chains do not fully participate in SC crystallization and instead remain in amorphous regions or form residual homocrystals. This mixed crystalline structure is consistent with the DSC results discussed in Section 3.3.

To further elucidate the nanoscale structural organization, SAXS profiles were analyzed (Figure 7b). The 50:50 blend exhibited a pronounced scattering feature in the low-q region, indicating enhanced electron density fluctuations associated with well-developed nanostructures. This behavior indicates enhanced nanoscale structural organization associated with stereocomplex-rich domains.

In contrast, neat PLLA and PDLA showed weak and diffuse scattering profiles, suggesting less organized internal structures dominated by homocrystalline and amorphous phases. The non-equimolar blends displayed intermediate scattering behavior, indicating partial structural ordering and nanoscale heterogeneity. The broader and less intense SAXS features suggest reduced structural regularity compared to the equimolar system.

From a structural perspective, the combined WAXD and SAXS results clearly demonstrate that stereocomplex formation leads to a hierarchical organization, spanning from crystal lattice (WAXD) to nanoscale packing (SAXS). The enhanced SC formation in the 50:50 blend results in a highly compact structure with reduced free volume and increased tortuosity, which is expected to restrict molecular diffusion pathways.

This structural densification is directly correlated with the improved functional properties observed in this study, including reduced water vapor permeability (Section 3.8) and enhanced resistance to microbial growth (Section 3.10). The combined DSC, WAXD, SAXS, and AFM results consistently demonstrate that stereocomplex formation induces hierarchical structural organization across multiple length scales. The enhanced crystallinity and compact nanoscale packing observed in the 50:50 blend reduce free volume and diffusion pathways, thereby contributing to improved barrier performance and microbial resistance.

### 3.6. Mechanical Properties

**Figure 8:**
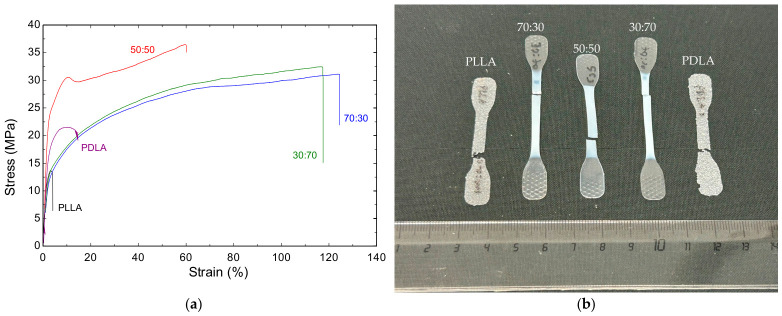
(**a**) Representative stress–strain curves from at least three independent measurements of PLLA, PDLA, and blends. (**b**) Fractured specimens after tensile testing.

The mechanical properties of PLLA, PDLA, and PLLA/PDLA blend films were evaluated by tensile testing, and the corresponding stress–strain curves are presented in Figure 8a, while the fractured specimens after testing are shown in Figure 8b. The quantitative tensile properties, including tensile strength, elongation at break, and Young’s modulus, are summarized in Table 5. All tensile properties reported in Table 5 represent the mean ± standard deviation obtained from at least three independent measurements.

Neat PLLA exhibited relatively low elongation at break and moderate tensile strength, indicating its brittle nature. Similarly, PDLA showed limited ductility, although with slightly improved elongation compared to PLLA. In contrast, the PLLA/PDLA blend films demonstrated significantly enhanced mechanical performance, particularly in terms of ductility and overall toughness [30].

Among the blends, the 50:50 composition exhibited the highest tensile strength (≈37 MPa) and Young’s modulus, which can be attributed to the formation of well-developed stereocomplex (SC) crystallites acting as physical crosslinking points within the polymer matrix [31,32]. These SC crystallites enhance intermolecular interactions and partially restrict chain mobility, resulting in improved load-bearing capability and stiffness.

Interestingly, the non-equimolar blends (70:30 and 30:70) showed higher elongation at break compared to the 50:50 blend, indicating improved ductility. This behavior is attributed to the presence of excess PLLA or PDLA chains that do not participate in stereocomplex formation. These unpaired chains remain in the amorphous phase or form homocrystalline domains, acting as soft segments that allow for greater chain mobility and energy dissipation during deformation. As a result, the materials can undergo more extensive plastic deformation before fracture, as evidenced by the elongated shapes of the fractured specimens in Figure 8b.

These findings demonstrate that the mechanical behavior of PLLA/PDLA blends is governed by the balance between stereocomplex crystallization and residual amorphous chain mobility. The equimolar blend favors strength and stiffness through efficient SC formation, whereas the non-equimolar blends provide enhanced flexibility and toughness due to partially constrained network structures. These results suggest that solvent-evaporation-controlled stereocomplexation enables tunable mechanical performance depending on the targeted packaging application.

Overall, the results demonstrate that the formation of stereocomplex crystallites significantly improves the mechanical properties of PLLA/PDLA blends. By adjusting the blend ratio, it is possible to tailor the mechanical behavior of the films, enabling the design of materials with optimized performance for specific applications.

### 3.7. FTIR Analysis

**Figure 9:**
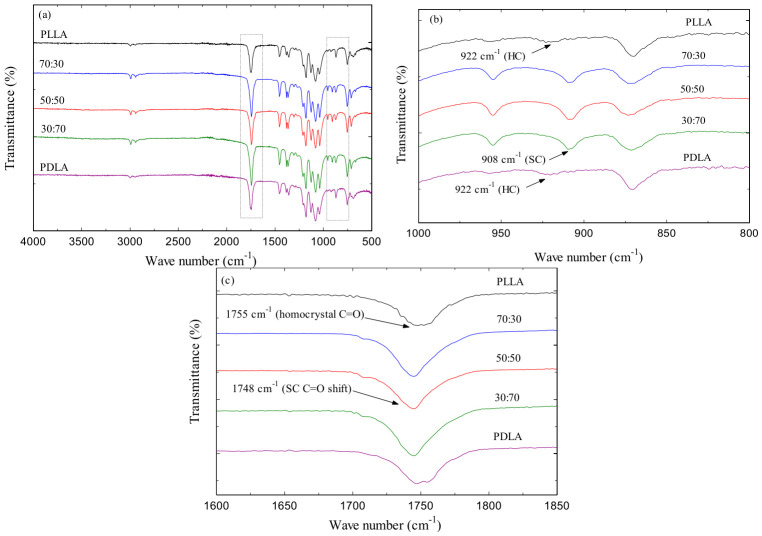
FTIR spectra of PLLA, PDLA, and blends highlighting SC-related spectral shifts and enhanced intermolecular interactions in PLLA/PDLA blend films: (**a**) full spectra, (**b**) fingerprint region highlighting SC and HC peaks at 908 and 922 cm^−1^, and (**c**) carbonyl stretching region showing a C=O shift from 1755 to 1748 cm^−1^ in the PLLA:PDLA blend.

Fourier-transform infrared (FTIR) spectroscopy was employed to investigate the molecular interactions and crystalline structures of PLLA, PDLA, and blends, as presented in Figure 9. The full spectra (Figure 9a) show that all samples exhibit a strong absorption band at approximately 1755 cm^−1^, corresponding to the C=O stretching vibration of ester groups in the PLA backbone, characteristic of the homocrystalline (HC) α-form [33].

In the PLLA/PDLA blends, particularly the equimolar 50:50 composition, this carbonyl band exhibits a slight shift toward lower wavenumbers (~1748 cm^−1^) along with noticeable peak broadening, as clearly observed in the magnified region (Figure 9c). This shift is attributed to enhanced intermolecular interactions between PLLA and PDLA chains, arising from the formation of stereocomplex (SC) crystallites. The change in peak position reflects a modified local chemical environment associated with stronger dipole–dipole interactions and tighter chain packing within the SC structure.

Additional evidence of stereocomplex formation is provided by the fingerprint region (1000–600 cm^−1^), as shown in Figure 9b. Neat PLLA and PDLA exhibit a dominant absorption band at approximately 922 cm^−1^, which is associated with HC crystalline structures. In contrast, the PLLA/PDLA blends display the emergence of a new band near ~908 cm^−1^, assigned to SC-specific vibrational modes [34]. The intensity of this SC-related band is most pronounced in the 50:50 blend, indicating the highest degree of stereocomplexation at the equimolar ratio.

Furthermore, absorption bands in the range of 1180–1080 cm^−1^, corresponding to C–O–C stretching vibrations, are present in all samples without significant shifts or intensity variations [34]. This suggests that the primary chemical structure of PLA remains unchanged, and that the observed spectral differences arise mainly from variations in crystalline organization rather than chemical modification. Moreover, as shown in Figure 9c, the observed shift in the carbonyl stretching band from 1755 to 1748 cm^−1^, together with the enhanced absorption intensity in the SC-sensitive region, indicates stronger intermolecular interactions and tighter chain packing between PLLA and PDLA chains. These spectral changes are consistent with the enhanced stereocomplex crystallization observed from DSC and WAXD analyses, which contributed to the improved mechanical strength and reduced water vapor permeability of the blend films.

Overall, the combined analysis of the full spectra (Figure 9a), fingerprint region (Figure 9b), and carbonyl stretching region (Figure 9c) provides consistent evidence for the formation of stereocomplex crystallites in PLLA/PDLA blends. These findings are in good agreement with the WAXD and SAXS results, confirming that stereocomplexation enhances intermolecular interactions and structural packing. Such structural evolution contributes to the improved mechanical properties and reduced permeability observed in the blends, making them promising candidates for advanced biodegradable packaging applications.

### 3.8. Water Vapor Permeability (WVP)

**Figure 10:**
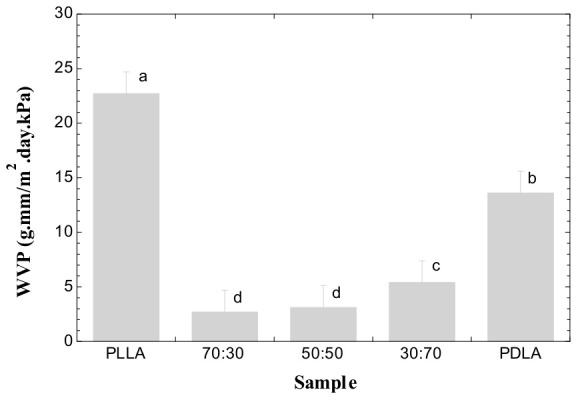
Water vapor permeability (WVP) of PLLA, PDLA, and PLLA/PDLA blend films. The blends exhibit significantly lower WVP compared to neat polymers, indicating enhanced barrier performance associated with stereocomplex formation. Different letters indicate statistically significant differences (*p* < 0.05).

The water vapor permeability (WVP) of the films was evaluated using the gravimetric method based on ASTM E96. As summarized in Table 6 and illustrated in Figure 10, the neat PLLA film exhibited the highest WVP value (22.7 g·mm/m^2^·day·kPa), indicating relatively poor barrier performance. In contrast, PDLA showed a moderate WVP value (13.6 g·mm/m^2^·day·kPa), suggesting a slightly improved resistance to water vapor transmission compared to PLLA [35].

Notably, all PLLA/PDLA blend films demonstrated significantly lower WVP values than the neat polymers, with values of 2.69, 3.11, and 5.39 g·mm/m^2^·day·kPa for the 70:30, 50:50, and 30:70 compositions, respectively [36]. The statistical analysis (*p* < 0.05) confirmed that the blends (group c) exhibited significantly reduced WVP compared to PLLA (group a) and PDLA (group b), highlighting the effectiveness of blending in enhancing barrier properties. Although the 70:30 blend exhibited the numerically lowest WVP value, the 50:50 blend showed the most balanced structure–property relationship, combining high stereocomplex crystallinity, reduced surface roughness, and consistently improved barrier performance. These results quantitatively confirm that enhanced stereocomplex formation leads to denser chain packing and more effective barrier performance.

The remarkable reduction in WVP for the blend films can be attributed to the formation of stereocomplex (SC) crystallites between PLLA and PDLA chains. These SC crystallites are known to possess higher crystallinity and stronger intermolecular interactions compared to homocrystals, resulting in a more compact and densely packed microstructure. Such structural densification effectively reduces the free volume and limits the diffusion pathways available for water vapor molecules.

Furthermore, the improved barrier performance of the blends is consistent with the morphological and structural analyses discussed in previous sections, where enhanced molecular packing and reduced surface roughness were observed. This suggests that barrier performance is governed not only by SC crystallinity, but also by chain packing heterogeneity, tortuosity, and residual amorphous phase distribution.

### 3.9. Degradation Behavior

**Figure 11:**
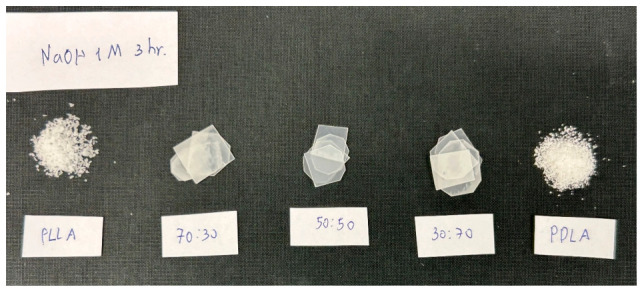
Photographs of PLLA, PDLA, and PLLA/PDLA blend samples after alkaline degradation in 1 M NaOH solution for 3 h.

**Figure 12:**
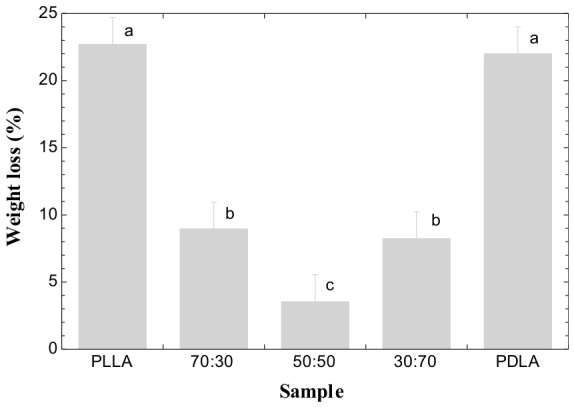
Weight loss (%) of PLLA, PDLA, and PLLA/PDLA blends after degradation in 1 M NaOH for 3 h. Different letters indicate significant differences (*p* < 0.05).

The degradation behavior of PLLA, PDLA, and PLLA/PDLA blend films was qualitatively evaluated after immersion in alkaline solution (NaOH, 1 M) for 3 h, as shown in Figure 11 and the corresponding weight loss values are presented in Figure 12. The visual observations reveal distinct differences in degradation resistance among the samples.

The neat PLLA and PDLA samples exhibited noticeable fragmentation and partial disintegration after treatment, indicating their relatively lower resistance to alkaline hydrolysis [37]. The degraded residues appeared as irregular particles or powder-like structures, suggesting extensive chain scission and structural breakdown.

In contrast, the PLLA/PDLA blend films demonstrated improved structural integrity after the same treatment [7,38]. Particularly, the 50:50 composition maintained a more coherent and compact morphology, with less fragmentation compared to the neat polymers. The 70:30 and 30:70 blends also showed enhanced resistance to degradation, although slight surface erosion and partial fragmentation were still observed.

The improved degradation resistance of the blend films can be attributed to the formation of stereocomplex (SC) crystallites between PLLA and PDLA chains. These SC crystallites possess higher crystallinity and stronger intermolecular interactions than homocrystals, resulting in a more tightly packed structure that hinders the penetration of hydroxide ions and slows down hydrolytic chain scission.

Moreover, the denser molecular packing in SC-rich regions reduces water uptake and limits diffusion pathways for degradation agents, contributing to the observed enhancement in stability [39]. Among the blends, the 50:50 composition exhibited the highest resistance, which is consistent with the expected maximum stereocomplex formation at equimolar ratios. The enhanced degradation resistance of the 50:50 blend is consistent with its higher stereocomplex crystallinity and denser microstructure, which reduce hydroxide ion diffusion into the film matrix. These results clearly demonstrate that stereocomplex formation not only improves thermal and barrier properties but also enhances the resistance of PLA-based materials to alkaline degradation, making them more suitable for applications requiring controlled durability.

The degradation behavior was further quantified by calculating the percentage weight loss after alkaline treatment. As shown, the PLLA/PDLA blends exhibited lower weight loss compared to the neat polymers, with the 50:50 composition showing the lowest value (~3.5%), indicating superior resistance to hydrolytic degradation. It should be noted that the present degradation study focused primarily on the initial degradation behavior under accelerated alkaline conditions to comparatively evaluate the influence of stereocomplex formation on film stability. Long-term degradation behavior under physiological or real storage environments requires further investigation and will be considered in future studies.

### 3.10. Microbial Growth Evaluation (Total Plate Count)

The antimicrobial performance of PLLA, PDLA, and PLLA/PDLA blend films was evaluated using the total plate count (TPC) method after storage with minced pork at 4 °C for 6 days. The microbial load was quantified and expressed as colony-forming units per gram (CFU/g), as summarized in Table 7.

**Figure 13:**
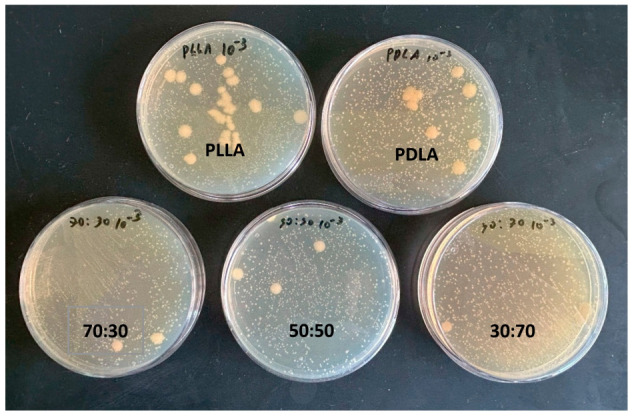
Total plate count (TPC) of samples after 6 days at a dilution of 10^−4^ using the spread plate method (0.1 mL), showing microbial growth on PLLA, PDLA, and PLLA/PDLA blend films.

**Figure 14:**
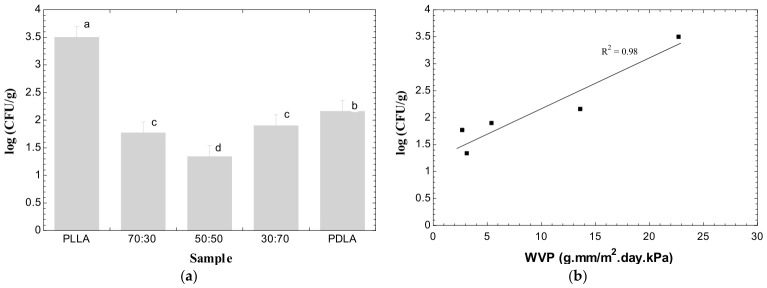
Microbial load of PLLA, PDLA, and PLLA/PDLA blend films after 6 days, presented as (**a**) log(CFU/g) and (**b**) correlation between water vapor permeability (WVP) and microbial load. Values represent mean ± standard deviation (*n* = 3). Different letters indicate statistically significant differences (*p* < 0.05).

As shown in Figure 13 and Figure 14, and Table 7, the neat PLLA film exhibited the highest microbial load, indicating relatively poor resistance to microbial proliferation (3.50 × 10^7^ CFU/g), followed by PDLA (2.16 × 10^7^ CFU/g). In contrast, all PLLA/PDLA blend films demonstrated reduced microbial growth compared to the neat polymers [40]. Among the blends, the 50:50 composition showed the lowest microbial load (1.34 × 10^7^ CFU/g), followed by the 70:30 (1.77 × 10^7^ CFU/g) and 30:70 (1.90 × 10^7^ CFU/g) samples.

The reduced microbial proliferation observed in the blend films can be primarily attributed to the enhanced barrier properties associated with stereocomplex (SC) crystallite formation. The SC structure is known to exhibit higher crystallinity and denser molecular packing compared to homocrystalline domains, which may limit moisture diffusion and reduce the availability of free volume for microbial growth. In particular, the 50:50 blend, which favors optimal SC formation at near-equimolar ratios, demonstrated the strongest resistance to microbial contamination.

Furthermore, a positive correlation between WVP and microbial load is observed (Figure 14b). Films with lower WVP values exhibit reduced microbial growth, highlighting the critical role of barrier properties in suppressing microbial proliferation. It should be noted that the present antimicrobial evaluation was based on total plate count (TPC) analysis, which reflects the overall microbial growth during storage rather than selective inhibition against specific bacterial strains. Therefore, the observed reduction in microbial proliferation is primarily attributed to the improved barrier properties and reduced moisture transfer associated with stereocomplex formation.

Overall, the results suggest that PLLA/PDLA stereocomplex films, especially at the 50:50 composition, are promising candidates for biodegradable food packaging applications, where improved resistance to microbial contamination is required.

## 4. Conclusions

This study demonstrated the critical role of solvent evaporation kinetics in governing the crystallization behavior and structure–property relationships of PLLA/PDLA blend films. Controlled slow solvent evaporation effectively promoted the formation of stereocomplex (SC) crystallites, particularly in the equimolar (50:50) composition, leading to a highly ordered and compact microstructure. Comprehensive structural analyses (DSC, WAXD, SAXS, and FTIR) consistently confirmed enhanced stereocomplex formation under controlled conditions. This structural evolution resulted in significantly improved functional properties, including higher tensile strength and stiffness, reduced surface roughness, and markedly enhanced barrier performance. In particular, the PLLA/PDLA blends exhibited a substantial reduction in water vapor permeability compared to neat polymers, highlighting the effectiveness of stereocomplexation in limiting molecular diffusion through dense crystalline domains. Furthermore, the formation of SC crystallites contributed to improved resistance against alkaline degradation and reduced microbial growth, indicating the multifunctional advantages of the blend films. Among all compositions, the 50:50 blend showed the most balanced and superior performance due to optimal stereocomplex formation. Overall, this work establishes solvent evaporation control as a simple yet powerful processing strategy for tailoring the microstructure and performance of PLA-based materials. The key novelty of this work lies not merely in stereocomplex formation itself, but in clarifying how solvent evaporation kinetics govern hierarchical structural evolution and multifunctional properties in PLLA/PDLA films. Overall, this work provides a simple and scalable strategy for tailoring biodegradable PLA films for advanced packaging applications.

## Figures and Tables

**Table 1:** Average diameter and thickness of PLLA/PDLA blend films.

Samples	Film Diameter (mm)	Film Thickness (mm)
PLLA	10.0 ± 0.3	0.38 ± 0.03
70:30	9.0 ± 0.3	0.45 ± 0.03
50:50	7.8 ± 0.3	0.52 ± 0.03
30:70	9.0 ± 0.2	0.45 ± 0.03
PDLA	10.0 ± 0.2	0.38 ± 0.03

**Table 2:** Thermal parameters and crystallinity of PLLA, PDLA, and PLLA/PDLA blend films prepared under open-lid and closed-lid conditions. Representative thermograms are presented, while all measurements were independently repeated to confirm reproducibility.

Sample	HC (Homocrystal)			SC (Stereocomplex Crystal)			*X_c,total_* (%)
	T_m,HC_ (°C)	Δ*H_m,HC_* (J/g)	*X_c,HC_* (%)	T_m,SC_ (°C)	Δ*H_m,SC_* (J/g)	*X_c,SC_* (%)	
Open-lid							
PLLA	174.40	49.88	53.63	-	-	-	53.63
70:30	172.34	13.14	14.13	225.32	38.46	27.08	41.21
50:50	175.20	3.52	3.78	225.15	55.14	38.80	42.58
30:70	164.86	6.71	7.21	227.34	30.24	21.30	28.51
PDLA	175.41	54.72	58.84	-	-	-	58.84
Closed-lid							
PLLA	173.46	50.94	54.77	-	-	-	54.77
70:30	163.66	6.56	7.05	221.18	66.74	47.00	54.05
50:50	-	-	-	221.63	72.38	50.97	50.97
30:70	166.83	4.36	4.69	222.34	29.30	20.63	25.32
PDLA	174.75	50.54	54.34	-	-	-	54.34

**Table 3:** Surface roughness parameters of PLLA, PDLA, and blends derived from AFM analysis. Values are presented as mean ± SD (*n* = 3).

Sample	Average Height (nm)	RMS Roughness (Sq) (nm)	Mean Roughness (Sa) (nm)	Maximum Height (Sz) (nm)
PLLA	51.60 ± 0.45	14.08 ± 0.14	11.36 ± 1.39	100.90 ± 10.21
70:30	24.31 ± 0.16	6.21 ± 0.55	4.90 ± 0.38	51.18 ± 0.47
50:50	27.01 ± 0.19	5.22 ± 0.47	4.12 ± 0.41	51.39 ± 0.54
30:70	40.77 ± 0.41	6.55 ± 0.56	4.77 ± 0.37	63.92 ± 0.63
PDLA	58.20 ± 0.35	15.72 ± 1.42	12.55 ± 1.11	106.10 ± 9.87

**Table 4:** Diffraction peak parameters (2*θ*, *q*, d-spacing) for SC and HC phases observed in PLLA/PDLA blends.

Phase	2*θ* (°)	*q* (nm^−1^)	d-Spacing (nm)	Assignment
SC	11.9	0.31	2.03	(110)
SC	20.7	0.63	1.00	(300)
HC	16.6	0.49	1.80	(200)/(100)
HC	18.8	0.66	1.51	(203)

**Table 5:** Mechanical properties of PLLA, PDLA, and PLLA/PDLA blends. Data are presented as mean ± SD (*n* ≥ 3).

Samples	Tensile Strength (MPa)	Elongation at Break (%)	Young’s Modulus (MPa)
PLLA	18.2 ± 0.5	8.5 ± 1.2	1150 ± 50
70:30	28.7 ± 0.7	124.5 ± 1.5	1350 ± 45
50:50	37.0 ± 0.8	62.1 ± 2.0	1450 ± 60
30:70	26.4 ± 0.6	120.7 ± 1.8	1300 ± 50
PDLA	22.4 ± 0.6	11.3 ± 1.1	1200 ± 40

**Table 6:** Water vapor permeability (WVP) of different films at 25 °C and 75% RH.

Samples	ΔWeight/Day (g/Day)	WVP (g·mm/m^2^·Day·kPa)
PLLA	0.10 ± 0.01	22.7 ± 2.0 ^a^
70:30	0.01 ± 0.00	2.69 ± 0.2 ^d^
50:50	0.01 ± 0.00	3.11 ± 0.3 ^d^
30:70	0.02 ± 0.00	5.39 ± 0.5 ^c^
PDLA	0.06 ± 0.00	13.6 ± 1.2 ^b^

Values are presented as mean ± SD (*n* = 3). Different letters indicate statistically significant differences (*p* < 0.05).

**Table 7:** Microbial load of PLLA, PDLA, and PLLA/PDLA blend films after 6 days.

Sample	Colony Count	CFU/g (×10^7^)
PLLA	350 ± 30 ^a^	3.5 ± 0.30 ^a^
70:30	177 ± 15 ^c^	1.77 ± 0.15 ^c^
50:50	134 ± 10 ^d^	1.34 ± 0.10 ^d^
30:70	190 ± 12 ^c^	1.90 ± 0.12 ^c^
PDLA	216 ± 19 ^b^	2.16 ± 0.19 ^b^

Values are presented as mean ± SD (*n* = 3). Different letters indicate statistically significant differences (*p* < 0.05).

## Data Availability

The original contributions presented in the study are included in the article; further inquiries can be directed to the corresponding author.
